# Fate of *Listeria monocytogenes*, *Salmonella* spp., and Shiga Toxin-Producing *Escherichia coli* on Slices of an All-Beef Soppressata during Storage

**DOI:** 10.3390/foods12101954

**Published:** 2023-05-11

**Authors:** John B. Luchansky, Laura E. Shane, Manuela Osoria, Bryan T. Vinyard, Bradley A. Shoyer, Stephen G. Campano, Anna C. S. Porto-Fett

**Affiliations:** 1 U.S. Department of Agriculture, Agricultural Research Service, Wyndmoor, PA 19038, USA; laura.shane@usda.gov (L.E.S.); manuela.osoria@usda.gov (M.O.); brad.shoyer@usda.gov (B.A.S.); anna.portofett@usda.gov (A.C.S.P.-F.); 2 U.S. Department of Agriculture, Agricultural Research Service, Beltsville, MD 20705, USA; bryan.vinyard@usda.gov; 3 Hawkins Inc., Roseville, MN 55113, USA; steve.campano@hawkinsinc.com

**Keywords:** beef soppressata, dry-cured meat, Shiga toxin-producing *Escherichia coli*, *Listeria monocytogenes*, *Salmonella*, charcuterie, shelf life

## Abstract

Cells of *Listeria monocytogenes*, *Salmonella* spp., or Shiga toxin-producing *Escherichia coli* (STEC) were inoculated (ca. 4.0 log CFU/slice) onto slices (ca. 4 g each slice) of an all-beef soppressata (ca. pH 5.05 and a_w_ 0.85). The storage of vacuum-sealed slices of inoculated soppressata at 4 °C or 20 °C for 90 days resulted in reductions of all three pathogens by ca. 2.2 to 3.1 or ca. ≥3.3 log CFU/slice, respectively. When pathogen levels decreased to below detection (≤1.18 log CFU/slice) by direct plating, it was possible to recover each of the target pathogens by enrichment, albeit more frequently from slices stored at 4 °C (*p* < 0.05) compared to 20 °C. In summary, the slices of the commercially produced beef soppressata selected for this study did not provide a favorable environment for either survival or outgrowth of surface-inoculated cells of *L. monocytogenes*, *Salmonella* spp., or STEC during storage.

## 1. Introduction

Prior to the availability of modern-day preservation technologies, such as refrigeration, freezing, pasteurization, high pressure processing, irradiation, and food-grade antimicrobials, which became mainstream only within the last 200 years or so, human beings were reliant on salting, drying, and/or fermentation to preserve meat [1,2]. Among the most popular of so-called “old-world-style” preserved meats are pork products from Southern Europe dating back to between 100 BC and 700 AD, such as dry-cured ham (e.g., prosciutto) and fermented salume/sausage (e.g., soppressata), respectively [2,3,4,5,6]. However, since pork is the primary protein species comprising these artisanal ready-to-eat (RTE) meats, such products cannot be universally enjoyed by all consumers, notably because some individuals adhere to a restrictive diet [7]. Thus, there has been considerable interest of late to utilize beef as the sole protein source in various specialty/ethnic meats that are traditionally made exclusively with pork. In addition to exploring and improving the taste and texture of artisanal meats made exclusively with beef, further research is also needed to establish and, if needed, to improve upon the safety of these products.

One example of a globally recognized, well-liked, Mediterranean-type salume is soppressata, a dry-cured, often flattened, salami made from coarsely ground leaner cuts of pork [8]. The process of preparing soppressata, namely fermentation and drying, is sufficient to both ensure safety and achieve shelf stability; however, on occasion, pathogens such as *Listeria monocytogenes*, *Salmonella* spp., and Shiga toxin-producing *Escherichia coli* (STEC) have been recovered from various Italian-type deli meats, and a handful of recalls and outbreaks have been linked to the consumption of fermented sausage, including soppressata, when contaminated with these pathogens [9,10,11,12,13,14,15,16,17,18,19,20]. Regarding recalls, as recently as 2023, there was a recall of ca. 70,000 pounds of RTE charcuterie products such as soppressata, Genoa, cappocolo, coppa, and calabrese due to potential contamination with *L. monocytogenes* [21]. An example of a somewhat recent US outbreak attributed to *L. monocytogenes* contamination of Italian-type deli meats is a listeriosis outbreak in 2021 across four states involving salami, mortadella, and prosciutto (12 cases, 1 death [22]). Likewise, there were two salmonellosis outbreaks in 2021 involving Italian-type deli meats: (i) illnesses caused by uncured prosciutto, soppressata, Milano salami, and coppa (produced by a single manufacturer) across 17 states (40 cases, 12 hospitalizations, 0 deaths [23]), and (ii) illnesses caused by salami sticks (produced by a single manufacturer) across 10 states (34 cases, 7 hospitalizations, 0 deaths [24]). Although there have not been any notable STEC illnesses in recent years in the US linked to Italian-type fermented sausage [25], such products were first identified as a “new” route of transmission for Shiga toxin-producing *E. coli* dating back to the mid-1990s [26]: The consumption of a dry-fermented sausage contaminated with serotype O157:H7 cells of *E. coli* sickened 17 people (3 hospitalizations) across 2 states in 1994 [27]. Note, STEC were also responsible for a 2007 outbreak in Denmark involving an organic beef sausage (20 cases, no cases of HUS, 0 deaths [28]). The association of the above-mentioned three pathogens with Italian-type fermented sausage, and on occasion with recalls and illnesses, is most likely due to inadequate processing, process deviations, improper handling, or post-process contamination [29].

Whereas much has been published on the prevalence and behavior of pathogens such as *L. monocytogenes*, *Salmonella* spp., and STEC in/on various Italian-type pork salumi, far less information has been published on the fate of these pathogens when associated with an otherwise similar all-beef salumi, inclusive of soppressata. Given the appreciable demand for all-beef artisanal meats in general, and for soppressata specifically, especially from individuals adhering to a halal or kosher diet, as well as given the occasional recovery of pathogens from traditional artisanal fermented/dried salami and attendant recalls and illnesses, there is both a need and an opportunity to collect scientifically sound data on the likelihood of products such as beef soppressata to support pathogen persistence and proliferation. Thus, in the present study, we monitored the fate of *L. monocytogenes*, *Salmonella* spp., or STEC on slices of a commercial all-beef soppressata during storage at both refrigeration and ambient temperatures.

## 2. Materials and Methods

### 2.1. Bacterial Strains

Individual slices of an all-beef soppressata were surface-inoculated, as elaborated below, with either a multi-strain cocktail of eight rifampicin-resistant strains of STEC (100 μg rifampicin/mL; TCI America, Portland, OR, USA), five rifampicin-resistant strains of *Salmonella* spp. (100 μg rifampicin/mL), or five rifampicin-resistant strains of *L. monocytogenes* (100 μg rifampicin/mL). These multi-strain pathogen cocktails (Table 1) were separately prepared, confirmed, cultured, and/or maintained as described previously [30].

### 2.2. Inoculation of Soppressata Slices

Multiple packages (142 g each, single chub per package) from different production lots of a single brand of a commercially produced all-beef soppressata (Brand A) were purchased from an online vendor. The outside of each chub was wiped down with a paper towel moistened with 70% alcohol before the collagen casing was removed with the aid of a sterile scalpel. Next, each chub was sliced (Model 8512, Univex, Salem, NH, USA), and then, a single slice (ca. 3.9 ± 0.34 g each; ca. 3.5 cm diameter; ca. 0.1 cm thick) of soppressata was placed into a cast nylon and polyethylene copolymer bag containing vinyl acetate (Item #185-10; 3 mil standard barrier, 15.2 by 20.3 cm; Associated Bag, Milwaukee, WI, USA). Each face of each slice was separately inoculated with a 25-µL portion (50 µL total; ca. 4.0 log CFU/slice) of overnight-grown (ca. 18–20 h) rifampicin-resistant cells of each pathogen cocktail. The bags containing inoculated slices of soppressata were vacuum-sealed to 950 mBar with an Ultravac 550 vacuum packaging unit (Koch Equipment, Kansas City, MO, USA) and then stored at 4 °C or 20 °C for 90 days. In each of the three trials, each of three bags containing an inoculated slice of soppressata was separately analyzed for viable cells of a target pathogen at each sampling interval (N = 3 trials; *n* = 3 inoculated slices per sampling interval).

### 2.3. Chemical Analyses

Proximate chemical analyses were conducted on a single ca. 400 g composite (representative) sample from each of the two batches/production lots for each beef salume product purchased online between May and August of 2022 (N = 2 trials of each brand of salume; *n* = 1 sample per each batch/lot of each product). Note, in addition to the brand of salume that we separately inoculated with the three pathogen cocktails (i.e., Brand A), for comparative purposes, we also elaborated the chemical composition of two additional all-beef salume products (Brand B and Brand C) that were not inoculated. Chemical analyses of all three brands of salume were conducted by an independent commercial testing facility using methods approved by the Association of Official Analytical Chemists [31] as follows: fat (AOAC 960.39), salt (AOAC 983.14), ash (AOAC 920.153), moisture (AOAC 950.46Bb), protein (AOAC 991.20.i), carbohydrates (by difference), sodium nitrite (AOAC 973.31), titratable acidity (AOAC 942.15), and water activity (AOAC 978.18).

### 2.4. Microbiological Analyses

Inoculated slices of soppressata were analyzed on days 0, 7, 15, 21, 28, 45, 60, 75, and/or 90 essentially as described [32]. In brief, after opening each bag with the aid of alcohol sterilized scissors, 15 mL of 0.1% sterile peptone water (Difco; Becton, Dickinson Company, Sparks, MD, USA) were added to each bag and the contents were massaged by hand for ca. 1 min. Portions of the resulting rinsate, with and without prior dilution in 0.1% peptone water, were plated onto sorbitol-MacConkey (SMAC; Difco), xylose-lysine-tergitol-4 (XLT-4; Difco), or modified Oxford (MOX; Difco) agar plates containing rifampicin (100 µg/mL) to recover surviving cells of STEC, *Salmonella* spp., or *L. monocytogenes*, respectively. The SMAC and XLT-4 agar plates were incubated at 37 °C for 24 h, whereas the MOX agar plates were incubated at 37 °C for 48 h. Colonies typical for each pathogen were enumerated and expressed as log CFU/slice. When pathogen levels decreased to below the detection limit (i.e., ≤1.18 log CFU/slice) by direct plating, pathogen presence was determined via enrichment as previously described [30]. In addition, at each sampling point, the pH of the rinsate was measured using a model 6000P pH/temperature electrode and a model 5500 pH meter (Daigger, Vernon Hills, IL, USA). As received from the online vendor, the 400 g composite salumi samples (see preceding section) were also separately analyzed for the presence/absence of cells of STEC (serogroups O26, O45, O103, O104, O111, O121, O145 (AOAC RI 09130) and serotype O157:H7 (AOAC RI 031002)), *L. monocytogenes* (AOAC 2003.12), and *Salmonella* spp. (AOAC 2003.09). The initial total aerobic plate count (APC; AOAC 966.23) and initial levels of lactic acid bacteria (LAB [33]) were also enumerated for each brand of salume.

### 2.5. Statistical Methods

Values for log CFU and pH values were analyzed by fitting a Pathogen (3) × Temperature (2) × Day (9) ANOVA model. The heterogeneity of within-treatment variability for these 54 treatments was incorporated into the model by assigning each treatment to a group. Treatments assigned to the same group had variances within four-times magnitude of all treatments in that group. Groups were specified as a random effect to ensure that the appropriate magnitude of within-treatment variability was associated with each treatment for all statistical tests and comparisons obtained from the ANOVA models. For proximate composition, one-way Sample (3) ANOVA models were fit for each test. All statistical analyses were conducted using SAS PROC MIXED [34] software, with α = 0.05 LSD pairwise means comparisons [35] obtained using SLICE = options in the LSMEANS statement.

## 3. Results and Discussion

As elaborated by others [36,37], meat preservation has transitioned from a necessity for survival/subsistence in ancient times to a luxury marked by convenience and unique sensory attributes in more recent times. One of the best examples of a modern-day indulgence is consumption of charcuterie trays comprised of artisanal meats [38,39,40]. The longstanding demand for high-quality and locally sourced artisanal meats and, in more recent years, especially for all-beef products formulated to accommodate individuals on a restrictive diet, have been the catalyst for the ongoing development of new types, tastes, and textures of meat products for consumers to enjoy. It has also served as a justification to scientifically validate the safety and to certify the shelf life of such products. As detailed elsewhere [41,42,43,44,45,46,47,48,49,50,51,52,53,54], our group and other investigators have quantified the potential for various all-beef meats that are dried, salted, cured, cooked, and/or fermented, inclusive of breakfast meats, savory snacks, and salted/dried and/or fermented products, to support the viability or outgrowth of various foodborne bacterial pathogens. Regardless of product categories and characteristics, the physicality and logistics of slicing and packaging, as well as extensive and unavoidable hand manipulation during the preparation and assembly of artisanal foods on charcuterie trays, provide ample opportunities for the introduction of food-borne pathogens onto the surface of RTE artisanal meats [55]. Such conditions also provide adequate justification for collecting additional data related to the occurrence, levels, and control of target pathogens on niche meat products that are fermented/dried, inclusive of all-beef products. Thus, the primary objective of the present study was to determine if an all-beef soppressata would allow for survival or support the (out)growth of *L. monocytogenes*, *Salmonella* spp., or STEC during storage.

To our knowledge, there have been no data published on the chemical composition of soppressata prepared with beef as the sole protein source. Thus, as one objective of this study, we elaborated the proximate composition of a commercial all-beef soppressata (Brand A) that was subsequently sliced and inoculated with cells of *L. monocytogenes*, *Salmonella* spp., or STEC (Table 2). For comparative purposes, we also quantified the proximate composition of two additional all-beef salumi products (i.e., Brand B and Brand C) that were also purchased online (Table 2). Chemical analyses revealed no significant (*p* > 0.05) differences in acidity, carbohydrates, fat, moisture, nitrite, protein, or pH levels among Brand A, Brand B, and Brand C. In contrast, chemical analyses revealed Brand C salume as discernibly (*p* < 0.05) different from Brand A and Brand B products with respect to salt content. Significant differences (*p* < 0.05) in ash levels among all three brands were also observed. Lastly, chemical analyses revealed that Brand C was similar (*p* > 0.05) to Brand A with respect to a_w_, but it had a significantly (*p* < 0.05) higher a_w_ when compared to Brand B salume. Note, no differences (*p* > 0.05) were observed between Brand A and Brand B products with respect to a_w_. In general, on average, the three brands of commercial all-beef salami displayed a slightly low pH (ca. pH 5.0) and low water activity value (ca. a_w_ 0.86), an intermediate moisture level (ca. 36%), and a relatively high fat (ca. 27%) and high salt (ca. 4.9%) content (Table 2). It should also be noted that there were no significant changes (*p* > 0.05) in pH of the inoculated slices of the all-beef soppressata (Brand A) after 90 days of storage at 4 °C or 20 °C; the initial pH was pH 4.97 ± 0.09, and regardless of the storage temperature, the final pH of the product after 90 days of storage was pH 5.15 ± 0.03. In comparison to compositional data for the all-beef soppressata analyzed herein, Liguori et al. [56] reported on the moisture (ca. 40%), protein (ca. 32%), and fat (ca. 25%) levels of a traditional pork-based soppressata prepared at an industrial establishment. As another example, Jung and colleagues [57] analyzed salchichón, a soppressata-like, dry-cured pork salume originating from Spain that displayed the following attributes after a 45-day ripening period: moisture (ca. 24%), protein (ca. 44%), ash (ca. 4.7%), fat (ca. 26%), a_w_ (ca. a_w_ 0.80), and pH (ca. pH 6.4). Romeo et al. [58] evaluated the chemical composition of typical Calabrian cured meats made from pork, including soppressata, and reported levels of about 4.1% ash, 27.9% fat, and 29.3% protein, with a water activity level of a_w_ 0.835 and ca. pH 5.6. Lastly, Coppola and colleagues [59] reported levels of about 35.8% moisture, 6.2% ash, 39% protein, 17.2% fat, and ca. pH 6.0 for an all-pork soppressata, as traditionally produced in Southern Italy. The above-mentioned peer-reviewed compositional data for soppressata-type salumi prepared with pork compare favorably with the data reported herein for a soppressata-type salume prepared exclusively with beef.

Retrospectively, and based solely on its chemical composition (Table 2), it is not likely that Brand A soppressata would support (appreciable) outgrowth of any of the three pathogens tested herein during cold storage for 90 days. In support of this statement and depending on the menstruum/substrate and temperature evaluated, *L. monocytogenes* can grow at salt levels of up to ca. 6.5% and at ≥pH 4.4, whereas most strains will not grow at ≤a_w_ 0.93 [60]. *Salmonella* can also grow at pH 4.0 to pH 4.5, but most strains are not able to grow at salt levels of ≥2.5% or at water activity values of ca. ≤a_w_ 0.93 when held at refrigeration temperatures [61,62]. For STEC, the reported minimum conditions for growth are pH 4.0 to pH 4.5, ≤a_w_ 0.89, and salt levels of ≤6.5% [61,63,64,65]. For point of reference, Brand A soppressata displayed a salt content of 5.43%, a water activity level of a_w_ 0.85, and an acidity/basicity factor of pH 5.05 (Table 2).

We also conducted a microbiological profile of the three commercial brands of salume as received directly from the attendant online vendors (Table 3). A single soppressata sample from each of two trials/batches from each of the three brands of all-beef salami all tested negative (<0.04 cells/g) by enrichment for *L. monocytogenes*, *Salmonella* spp., and STEC. In addition, significantly (*p* < 0.05) higher levels of LAB were observed for Brand A and Brand C salami compared to Brand B salume; levels of LAB ranged from <1 cell/g for Brand B to ca. 5.1 log CFU/g for Brand A and Brand C salami (Table 3). Although it remains unclear why levels of LAB were appreciably lower for Brand B salume, it is possible that factors such as product age (e.g., use by date), differences in levels and types of autochthonous LAB on raw materials, and/or differences in processing parameters could collectively account for such divergences. Variations were also observed among brands related to results for APC, with appreciably higher levels (*p* < 0.05) observed for Brand A when compared to Brand B salume; however, no significant (*p* > 0.05) differences in APC levels were observed between Brand C compared with Brand A or Brand B products; levels of APC ranged from ca. 1.2 to 5.5 log CFU/g (Table 3).

**Figure 1:**
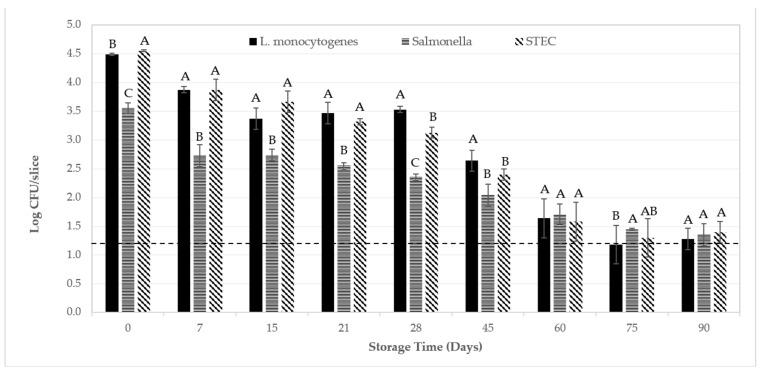
Recovery of *Listeria monocytogenes*, *Salmonella* spp., and Shiga toxin-producing *Escherichia coli* (STEC) (log CFU/slice) inoculated onto slices of beef soppressata during extended storage at 4 °C. Error bars represent the standard error of the mean (N = 3, *n* = 3). The dotted line (-------) denotes the detection limit of ≤1.18 log CFU/slice. When *L. monocytogenes*, *Salmonella* spp., or STEC numbers were below the detection limit, no cells of any of the three pathogens tested were recovered via enrichment. For a given storage day, bars/means that have no uppercase letter in common denote statistical differences (α = 0.0001) among pathogens.

**Figure 2:**
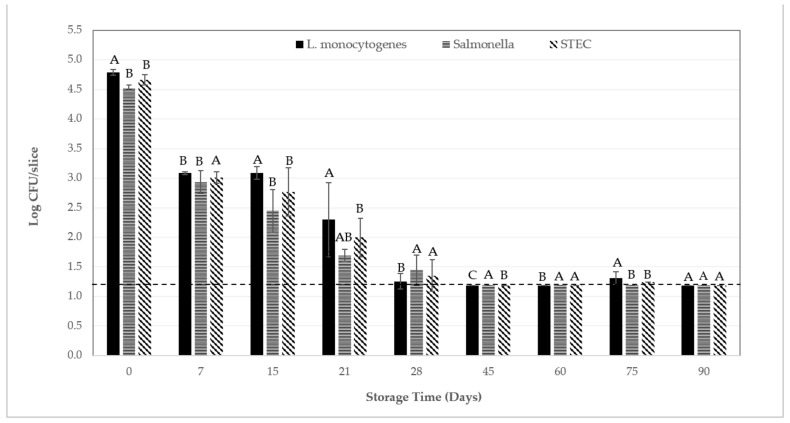
Recovery of *Listeria monocytogenes*, *Salmonella* spp., and Shiga toxin-producing *Escherichia coli* (STEC) (log CFU/slice) inoculated onto slices of beef soppressata during extended storage at 20 °C. Error bars represent the standard error of the mean (N = 3, *n* = 3). The dotted line (-------) denotes the detection limit of ≤1.18 log CFU/slice. When *L. monocytogenes*, *Salmonella* spp., or STEC numbers were below detection limit, no cells of any of the three pathogens tested were recovered via enrichment. For a given storage day, bars/means that have no uppercase letter in common denote statistical differences (α = 0.0001) among pathogens.

It has been well established that *L. monocytogenes*, *Salmonella* spp., and STEC are recoverable from a variety of Italian-type sausage. For example, De Cesare and collaborators [66] recovered cells of *L. monocytogenes* from ca. 15% (36 of 237 samples) of fermented sausage produced in Northern Italy; pathogen levels in positive samples were estimated at <10 MPN/g. Likewise, Quaglia et al. [67] recovered cells of *L. monocytogenes* from ca. 6.5% (2 of 31 samples) of pork soppressata samples produced in Southern Italy; pathogen levels in positive samples were estimated at ca. 1.5 to 1.8 log CFU/g. Note that these same soppressata samples also tested negative for cells of serotype O157:H7 and serotype O26 strains of *E. coli*. In another study, Hussein and Bollinger [68] summarized that the prevalence of serotype O157:H7 and non-O157:H7 cells of STEC in raw and fresh sausage worldwide, inclusive of beef sausage, ranged from 0.1 to 4.4% and 17 to 49.2%, respectively; pathogen levels in positive samples were not reported. Regarding *Salmonella* spp., Levine et al. [69] reported a 3-year cumulative prevalence of 1.43% (10 of 698 samples) for dry and semi-dry sausage collected in the late-1990s in the US from ca. 1800 federally inspected facilities; pathogen levels in positive samples were not reported. As another example, Cabedo and colleagues [70] reported the recovery of cells of *Salmonella* spp. from 9 of 81 (11.1%) cured/dried pork sausage samples collected between 1998 and 2004 from retail stores or from food processing facilities in Spain; pathogen levels in positive samples were not reported. It has also been well established that cells of *L. monocytogenes*, *Salmonella* spp., and STEC have, on occasion, caused human illness from consumption of fermented sausage when/if contaminated with these pathogens by whatever means or routes available. As such, it seems probable that cells of these pathogens, if present on the raw materials or when associated with any of the ingredients used to prepare salami, might remain on the finished product and/or that post-process contamination would exacerbate the risk of food-borne illness. Therefore, from a public health perspective, it is significant that slices of the commercial all-beef soppressata tested herein did not provide a favorable environment for the outgrowth or survival of *L. monocytogenes*, *Salmonella* spp., or STEC during extended storage at 4 °C or 20 °C: levels of all three pathogens were reduced by ca. 2.2 to 3.3 log CFU/slice within 90 days (Figure 1 and Figure 2).

When inoculated slices of soppressata were stored at 4 °C, appreciable differences (*p* > 0.05) in pathogen recovery via direct plating were not observed; initial levels of all three pathogens decreased to ca. 1.4 log CFU/slice after 90 days (Figure 1). However, due to the lower (*p* < 0.05) initial levels of *Salmonella* spp. (3.6 log CFU/g) used to the inoculate slices of soppressata compared to the initial levels of *L. monocytogenes* or STEC (ca. 4.5 log CFU/slice), it was not possible to quantify a 3.0-log reduction for *Salmonella* by direct plating (detection limit of ≤1.18 log CFU/slice). Regardless, all three pathogens were recovered by direct plating from at least some slices of soppressata at each sampling interval during storage at 4 °C. Overall, although only subtle differences in the levels of the three pathogens were observed during refrigerated storage, there were no significant (*p* > 0.05) differences in levels of *Salmonella*, *L. monocytogenes*, or STEC after 90 days of storage at 4 °C (Figure 1). That being said, when levels of *L. monocytogenes* decreased to below the detection limit by direct plating beginning on day 75 (6 of 9 samples negative by direct plating) but also on day 90 (1 of 9 samples negative by direct plating), all 7 of these samples testing negative by direct plating subsequently tested negative by enrichment as well (Table 4). For *Salmonella* spp., when pathogen levels on some slices of soppressata decreased to below detection by direct plating beginning on day 60 (1 sample negative by direct plating) but also on day 75 (2 samples negative by direct plating) and on day 90 (4 samples negative by direct plating), 4 of these 7 (57%) samples testing negative by direct plating subsequently tested positive by enrichment (Table 4). Likewise, for STEC, when pathogen levels on some slices of soppressata tested negative by direct plating beginning on day 60 (1 sample negative by direct plating) but also on day 75 (6 samples negative by direct plating) and on day 90 (4 samples negative by direct plating), 4 of these 11 (36%) samples testing negative by direct plating subsequently tested positive by enrichment (Table 4).

When inoculated slices of soppressata were stored at 20 °C, subtle but significant (*p* < 0.05) differences in pathogens levels were observed throughout storage (Figure 2); however, appreciable differences in recovery among pathogens were not observed (*p* > 0.05) after 90 days of storage at 20 °C (Figure 2). In general, initial starting levels of all three pathogens were reduced from ca. 4.6 log CFU/slice to ca. 1.3 to 1.7 log CFU/slice within 28 days during storage at 20 °C. However, when levels of *L. monocytogenes* on some slices of soppressata decreased to below detection by direct plating beginning on day 21 but also on day 28 through to day 90, only 2 of 39 (5%) of such samples subsequently tested positive by enrichment (Table 4). When levels of *Salmonella* spp. on some slices of soppressata decreased to below detection via direct plating beginning on day 28, and continuing from day 45 through day 90, 17 of 38 (45%) of such samples subsequently tested positive by enrichment (Table 4). Likewise, when levels of STEC on some slices of soppressata decreased below the detection limit beginning on day 21, and continuing through day 90, 8 of 38 (21%) of such samples subsequently tested positive by enrichment (Table 4). 

There have been only a handful of recalls and illnesses attributed to soppressata-type products, presumably because the intrinsic attributes of such products typically do not provide a favorable environment for persistence or proliferation of *L. monocytogenes*, *Salmonella* spp., or STEC over the anticipated refrigerated shelf life [47]. It has also been well established that fermentation and drying alone will typically deliver only a ≤2.0 log reduction in levels of *L. monocytogenes*, *Salmonella* spp., or STEC [71,72,73,74,75,76]. Numerous publications have detailed the occurrence and fate of *L. monocytogenes*, *Salmonella* spp., or STEC during the manufacture and storage of Italian-type, dry-cured salume containing pork, but relatively few studies are specific to soppressata. As one example of the latter, Luchansky and colleagues [47] monitored the fate of *L. monocytogenes* or STEC in soupie, an old-world-style, specialty/ethnic pork soppressata, during fermentation, drying, and storage at 20 °C; reductions of ca. ≥5.0 log in levels of both pathogens were observed after 4 and 1 month, respectively, during storage. Regarding the fate of *Salmonella* during production of Italian salami, Bonilauri and colleagues [77] reported reductions ranging from ca. 1.0 to 4.5 log CFU/g.

The literature cited herein confirms that a traditional pork-based soppressata does not support survival of *L. monocytogenes*, *Salmonella* spp., or STEC during manufacture or storage. Similarly, the present study provides the first scientifically sound data confirming that an otherwise similar soppressata made from beef rather than pork does not provide a favorable environment for viability or persistence of these same three pathogens during storage. More specifically, storage of inoculated slices of beef soppressata at 4 °C or 20 °C resulted in reductions of 2.2 to 3.3 log CFU/slice in levels of *L. monocytogenes*, *Salmonella* spp., or STEC. Our data also established that when levels of *L. monocytogenes*, *Salmonella* spp., or STEC decreased to below detection by direct plating (≤1.18 log CFU/slice), it was more likely on occasion to separately recover all three target pathogens by enrichment from vacuum-packaged slices of beef soppressata following storage at 4 °C (*p* < 0.05) compared to 20 °C. Nonetheless, our validation of the wholesomeness of (slices of) an all-beef soppressata confirms the safety and broadens the market for this category of dry-cured, Italian-type salumi and interpolates the food preferences of people adhering to a restrictive diet.

## Figures and Tables

**Table 1:** Strains of *L. monocytogenes*, *Salmonella* spp., and Shiga toxin-producing *Escherichia coli* (STEC) used in this study.

Bacterial Strains	Strain Designation	Source	Type ^1^
*L*. *monocytogenes*	MFS2	Environmental isolate from a pork processing plant	1/2a
	H7776	Frankfurter isolate, 1998 outbreak	4b
	ScottA	Clinical isolate, 1983 Massachusetts pasteurized milk outbreak	4b
	LM-101M	Beef and pork sausage isolate	4b
	F6854	Turkey frankfurter isolate	1/2a
*S*. *Typhimurium*	MFS 248	Hog carcass isolate	
*S*. *Typhimurium*	MFS 330	Hog carcass isolate	
*S*. *Typhimurium*	FSIS OB060362	Clinical isolate associated with pork sausage	
*S*. *Typhimurium*	H3380	Clinical isolate	DT104
*S*. *Copenhagen*	MFS 3446	Pork isolate	
STEC	H30	Infant with diarrhea	O26:H11
	CDC 96-3285	Human stool	O45:H2
	CDC 90-3128	Human stool	O103:H2
	ATCC BAA-2326	Human stool	O104:H4
	JB1-95	Clinical isolate	O111:H-
	CDC 97-3068	Human stool	O121:H19
	83-75	Human stool	O145:NM
	USDA FSIS 011-82	Meat isolate	O157:H7

^1^ Serotypes for *L. monocytogenes* and STEC strains; phage types for *Salmonella* spp. strains.

**Table 2:** Proximate composition of all-beef salumi purchased from online vendors ^1^.

Analyte	Brand A ^2^	Brand B ^2^	Brand C ^2^	Average All Brands
Ash (%)	6.90 ± 0.18 ^3,A^	6.48 ± 0.05 ^B^	5.20 ± 0.03 ^C^	6.19 ± 0.88
Carbohydrates (%)	2.85 ± 0.70 ^A^	3.38 ± 1.78 ^A^	1.13 ± 0.74 ^A^	2.45 ± 1.18
Fat (%)	26.34 ± 5.03 ^A^	32.07 ± 4.04 ^A^	23.77 ± 0.42 ^A^	27.39 ± 4.25
Moisture (%)	35.51 ± 2.85 ^A^	30.66 ± 5.55 ^A^	42.06 ± 0.85 ^A^	36.07 ± 5.72
Protein (%)	28.42 ± 1.29 ^A^	27.42 ± 0.33 ^A^	27.85 ± 1.20 ^A^	27.90 ± 0.50
Salt (%)	5.43 ± 0.17 ^A^	5.27 ± 0.08 ^A^	4.02 ± 0.02 ^B^	4.90 ± 0.77
Nitrite (ppm)	<5.0 ppm ± 0.0 ^A^	<5.0 ppm ± 0.0 ^A^	<5.0 ppm ± 0.0 ^A^	<5.0 ppm ± 0.0
Acidity (%; as lactic acid)	2.95 ± 0.91 ^A^	2.39 ± 0.63 ^A^	2.54 ± 0.07 ^A^	2.62 ± 0.29
Water activity (a_w_)	0.850 ± 0.007 ^AB^	0.825 ± 0.034 ^B^	0.911 ± 0.006 ^A^	0.86 ± 0.04
pH	5.05 ± 0.08 ^A^	5.02 ± 0.16 ^A^	4.92 ± 0.02 ^A^	5.00 ± 0.07

^1^ Data are the average of the results for each analyte from analyses of a single ca. 400 g composite representative sample of soppressata (prior to inoculation) from each of two trials/batches purchased online for each of the three brands of all-beef salume (N = 2, *n* = 1). Within a row, means that have no letters in common are significantly different (*p* > 0.05) as determined by the Bonferroni LSD technique. ^2^ Ingredients from label: Brand A = beef, salt, natural flavoring, cultured celery powder, dextrose, spices, maltodextrin, yeast extract, potassium chloride salt, sea salt, starter culture; brand B = beef, sea salt, sauterne wine, contains less than 2% of the following: cane sugar, spices, natural flavoring, garlic, natural smoke flavoring, lactic acid starter culture; brand C = beef meat, salt, dextrose, celery juice, spices, cherry juice powder, garlic, lactic acid starter. Contains celery. ^3^ Average ± standard deviation.

**Table 3:** Microbiological profile of commercially prepared all-beef salumi ^1^.

Microorganism	Brand A	Brand B	Brand C
Aerobic plate count	5.40 ± 1.41 ^A^	1.22 ± 0.37 ^B^	2.77 ± 0.22 ^AB^
Lactic acid bacteria	5.01 ± 0.55 ^A^	<1 log/g ^2,B^	5.22 ± 0.02 ^A^
Shiga toxin-producing *Escherichia coli* O157:H7	Negative	Negative	Negative ^3^
Shiga toxin-producing *Escherichia coli* “Top Six” ^4^	Negative	Negative	Negative
*Listeria monocytogenes*	Negative	Negative	Negative
*Salmonella* spp.	Negative	Negative	Negative

^1^ Data are the average of the results for analyses of a single ca. 400 g composite representative sample of soppressata (prior to inoculation) from each of two trials/batches purchased online for each of the three brands of all-beef salume (N = 2, *n* = 1). Within a row, means that have no letters in common are significantly different (*p* > 0.05) as determined by the Bonferroni LSD technique. ^2^ Less than 0.04 cells/g. ^3^ Less than 10 cells/g. ^4^ Shiga toxin-producing *Escherichia coli* serogroups O26, O45, O103, O111, O121, and O145 (also known as “Top Six”).

**Table 4:** Recovery of *Listeria monocytogenes*, *Salmonella* spp., or Shiga toxin-producing *Escherichia coli* (STEC) by direct plating and by enrichment of inoculated beef soppressata slices after storage at 4 °C or 20 °C (N = 3, *n* = 3).

	Storage Temperature											
	4 °C						20 °C					
Storage Days	*L. monocytogenes*		*Salmonella*		STEC		*L. monocytogenes*		*Salmonella*		STEC	
	DP ^1,3^	E ^2,3^	DP	E	DP	E	DP	E	DP	E	DP	E
0	9/9 ^4^	0/0 ^5^	9/9	0/0	9/9	0/0	9/9	0/0	9/9	0/0	9/9	0/0
7	9/9	0/0	9/9	0/0	9/9	0/0	9/9	0/0	9/9	0/0	9/9	0/0
15	9/9	0/0	9/9	0/0	9/9	0/0	9/9	0/0	9/9	0/0	9/9	0/0
21	9/9	0/0	9/9	0/0	9/9	0/0	8/9	0/1	9/9	0/0	6/9	3/3
28	9/9	0/0	9/9	0/0	9/9	0/0	1/9	0/8	7/9	2/2	8/9	1/2
45	9/9	0/0	9/9	0/0	9/9	0/0	0/9	0/9	0/9	9/9	3/9	1/6
60	9/9	0/0	8/9	0/1	8/9	0/1	0/9	0/9	0/9	4/9	0/9	3/9
75	3/9	0/6	7/9	2/2	3/9	2/6	6/9	2/3	0/9	1/9	0/9	0/9
90	8/9	0/1	5/9	2/4	5/9	2/4	0/9	0/9	0/9	1/9	0/9	0/9

^1^ DP = direct plating. ^2^ E = enrichment. ^3^ Enrichment and direct plating results for beef soppressata slices stored at 4 °C or 20 °C for 90 days (three slices per each of three trials for a total of nine slices for each sampling day). ^4^ Number of slices from which cells of *L. monocytogenes*, *Salmonella* spp., or STEC were recovered by direct plating/total number of slices direct plated. ^5^ Number of slices from which cells of a given pathogen were recovered by enrichment/total number of slices enriched.

## Data Availability

The data presented in this study are available upon request from the corresponding author. These data are not publicly available due to the proprietary nature of the source/formulation of the substrates evaluated.
